# Neutrophils isolated from systemic lupus erythematosus patients exhibit a distinct functional phenotype

**DOI:** 10.3389/fimmu.2024.1339250

**Published:** 2024-03-08

**Authors:** Neelakshi R. Jog, Catriona A. Wagner, Teresa Aberle, Eliza F. Chakravarty, Cristina Arriens, Joel M. Guthridge, Judith A. James

**Affiliations:** ^1^ Arthritis and Clinical Immunology, Oklahoma Medical Research Foundation, Oklahoma City, OK, United States; ^2^ Departments of Medicine and Pathology, University of Oklahoma Health Science Center, Oklahoma City, OK, United States

**Keywords:** neutrophils, systemic lupus erythematosus, autoimmunity, cytokines, neutrophil extracellular traps

## Abstract

Neutrophil dysregulation, particularly of a low-density subset, is associated with systemic lupus erythematosus (SLE); however, the exact role of normal-density neutrophils in SLE remains unknown. This study compares activation and functional phenotypes of neutrophils from SLE patients and healthy controls to determine potential contributions to SLE pathogenesis. Surface activation markers and release of neutrophil extracellular traps (NETs), granule proteins, and cytokines/chemokines were measured in resting and stimulated neutrophils from SLE patients (n=19) and healthy controls (n=10). Select miRNA and mRNA involved in neutrophil development and function were also measured. Resting SLE neutrophils exhibited fewer activation markers compared to control neutrophils, and activation markers were associated with different plasma cytokines/chemokines in SLE patients compared to healthy controls. However, activation markers increased similarly in SLE and control neutrophils following stimulation with a TLR7/8 agonist, neutrophil growth factors, and bacterial mimic. At the resting state, SLE neutrophils produced significantly more CXCL10 (IP-10), with trends toward other increased cytokines/chemokines. Following stimulation, SLE neutrophils produced fewer NETs and proinflammatory cytokines compared to control neutrophils but more MMP-8. In addition, SLE neutrophils expressed less miR130a, miR132, miR27a, and miR223. In conclusion, SLE neutrophils exhibit distinct functional responses compared to control neutrophils. These functional differences may result from differential gene expression via miRNAs. Furthermore, the differences in functional phenotype of SLE neutrophils suggest that they may contribute to SLE differently dependent on the inflammatory milieu.

## 1 Introduction

Neutrophils are rapid-acting, short-lived cells, forming the first line of defense against invading microbes through various processes, including producing cytokines, releasing granules containing antimicrobial proteins (i.e., lactoferrin) and proteases (i.e., matrix metalloproteinases [MMPs]), and generating neutrophil extracellular traps (NETs). Neutrophil dysregulation, particularly increased NETosis, is linked to autoimmune diseases like systemic lupus erythematosus (SLE). In particular, a proinflammatory neutrophil subset termed low-density granulocytes (LDGs) is elevated in SLE patients (1). LDGs are transcriptionally distinct, producing more IFNγ, TNFα, type I IFNs, and NETs compared to autologous and healthy control normal-density neutrophils (referred to here as traditional neutrophils [TNs]) (2–4), suggesting possible mechanisms by which LDGs influence SLE pathogenesis.

In contrast to LDGs, NET formation is similar between SLE and healthy TNs (4), suggesting that TNs might exert different functional responses or not contribute to SLE pathogenesis. However, studies on the role of TNs in SLE pathogenesis are limited and somewhat conflicting. For example, one study suggested that TNs are pathogenic through the upregulation of IFNα mRNA (2), while another found that neutrophils may be regulatory by restricting CD4+ T cell proliferation (5). This study compares the activation and functional responses of SLE and healthy TNs to understand their contributions to SLE pathogenesis.

## 2 Methods

### 2.1 Patients

SLE patients and healthy controls were recruited at the Oklahoma Medical Research Foundation (OMRF) through the Oklahoma Rheumatic Disease Research Cores Center. All patients met the American College of Rheumatology (ACR) and ACR/EULAR criteria for SLE classification (6, 7), and all participants provided written informed consent before inclusion in the study. All studies were performed in accordance with the Helsinki Declaration and approved by the OMRF Institutional Review Board.

### 2.2 Neutrophil isolation

Neutrophils were isolated from whole blood using dextran sedimentation as previously described (8). Briefly, blood was collected in vacutainers with citrate anti-coagulant, and peripheral blood mononuclear cells were separated by density gradient centrifugation. Red blood cells were sedimented using dextran. Residual red blood cells in supernatants were lysed and enriched TNs were used for experiments. Neutrophil enrichment resulted in >95% viability and >97% purity.

### 2.3 Flow cytometry

Neutrophils were cultured with HBSS with calcium and magnesium + 1% BSA (HBSS++) alone (unstimulated control) or with 10µM R848 (InvivoGen), 0.1µg/ml G-CSF (Peprotech Inc), or 0.1µg/ml G-CSF + GM-CSF for 30 minutes or 300nM of the bacterial mimic formyl methionine leucine phenyl-alanine (fMLF, Sigma) for 3 minutes. Cells were stained with CD66b-PerCP-Cy5.5, CD11b-FITC, CD62L-BV650, and CD35-PE antibodies (all from Biolegend) for 20 minutes on ice. After washing, cells were fixed in 1% paraformaldehyde. Data were acquired with LSRII or FACSCelesta (BD Biosciences) cytometers and analyzed using FlowJo Software (Tree Star).

### 2.4 Neutrophil and plasma soluble mediators

Neutrophils were cultured for 3h in HBSS++ alone (unstimulated) or with R848 or a combination of G-CSF and GM-CSF (as above). Supernatants were collected by centrifugation, aliquoted, and stored at -20°C. Neutrophil granule proteins and cytokines, as well as plasma cytokines, were measured using custom xMAP assays (R&D systems) as previously described (9). Data were acquired on the BioPlex200 array system (Bio-Rad Technologies, Hercules, CA). Plates with >60% of samples below the standard range were excluded from subsequent analyses. Plasma levels of BLyS and APRIL were measured by ELISA according to the manufacturer’s instructions (R&D Systems).

### 2.5 NETosis

Neutrophils were seeded on poly-L Lysine coated chambered cover glass (Nunc/Labtek, 50,000 cells/chamber) with or without 10µM R848 or 0.1µg/ml G-CSF+GM-CSF for 4h, followed by fixation with 4% paraformaldehyde for 30min. After permeabilization with 0.25% triton X-100, cells were blocked with blocking buffer (5% goat serum in 1x PBS), and incubated overnight with polyclonal anti-MPO (Dako) and monoclonal anti-histone (Millipore) in blocking buffer at 4°C. Cells were stained with Rhodamine Red conjugated anti-rabbit and FITC conjugated anti-mouse antibodies. DNA was visualized by DAPI. NETs were visualized using a Zeiss LSM 710 confocal microscope, and data were analyzed with ImageJ.

### 2.6 miRNA and mRNA quantitation

Total RNA was extracted from neutrophils using a Quick RNA miniprep kit (Zymo Research). For miRNA quantitation, 10ng total RNA was reverse transcribed and pre-amplified using Qiagen miScript II RT and PreAmp kits. miRNA was quantitated using miScript SYBR Green PCR Kit and miScript Primer Assays on an ABI 7900HT thermal cycler. Total RNA was also used to quantify select mRNA expression using Fluidigm Biomark HD and delta gene assays per the manufacturer’s instructions (**Supplementary Table 1**).

### 2.7 Statistical analyses

For all analyses with 2 groups, comparisons were made using a 2-tailed Mann-Whitney test. A 2-way ANOVA with Sidak’s multiple comparisons test was used for analyses with 2 independent groups. Spearman’s rank correlation was used for correlation analyses. Heatmaps were created using Complexheatmap v.4.2 in R. All other analyses were performed using GraphPad Prism 9, and p-values less than 0.05 were considered statistically significant.

## 3 Results

### 3.1 Traditional neutrophils from SLE patients exhibit a reduced basal activation phenotype

**Figure 1:**
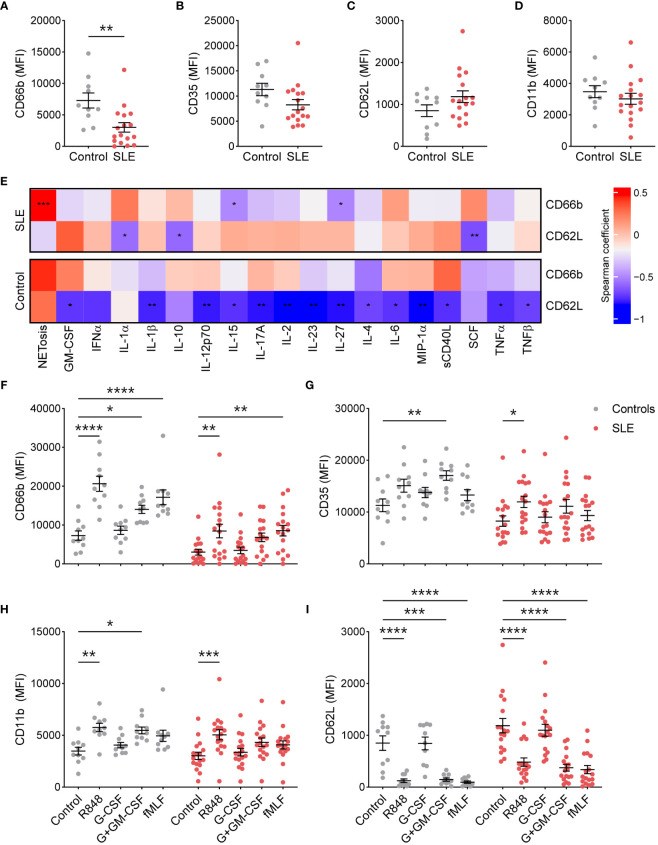
SLE traditional neutrophils are less activated at the resting state, but maintain their ability to respond to external stimuli. **(A)** CD66b, **(B)** CD35, **(C)** CD62L, and **(D)** CD11b expression was determined on traditional neutrophils isolated from controls or SLE patients using flow cytometry. Statistical significance was determined using a Mann-Whitney test. **(E)** Heatmap of Spearman’s rank correlation coefficients for plasma soluble mediator concentrations (MFI fold-change over control) that were significantly associated with CD66b or CD62L expression on traditional neutrophils isolated from controls or SLE patients. **(F)** CD66b, **(G)** CD35, **(H)** CD11b, and **(I)** CD62L expression was determined on unstimulated or stimulated traditional neutrophils isolated from controls or SLE patients using flow cytometry. Statistical significance was determined using a 2-way ANOVA with Sidak’s multiple comparisons test. Lines indicate mean+SEM. *p<0.05, **p<0.01, ***p<0.001, ****p<0.0001.

To determine whether TNs from SLE patients are more activated due to the existing inflammatory milieu, we determined the basal surface expression of neutrophil activation markers by flow cytometry. Surprisingly, compared to healthy controls (n=10), TNs from SLE patients (n=19) exhibited significantly lower expression of CD66b (**Figure 1A**), specifically in patients with low disease activity (**Supplementary Figure 1**). In addition, there was a trend towards reduced CD35, particularly in those with high disease activity, and increased CD62L expression compared to those from controls (**Figures 1A–C**, **Supplementary Figure 1**). CD11b expression was similar in TNs from SLE patients and healthy controls (**Figure 1D**). These data suggest that TN from controls and patients with high disease activity have similar baseline activation; however, TNs from low disease activity patients may have suppressed basal activation. The expression of activation markers did not differ based on corticosteroid usage (**Supplementary Figure 2**). Consistent with differing inflammatory environments, activation markers were associated with different plasma cytokines/chemokines in SLE patients compared to healthy controls (**Figure 1E**). In particular, neutrophil activation positively correlated with the expression of SCF in SLE patients but not controls.

Excessive stimulation, as expected in SLE, may result in neutrophil exhaustion, which could contribute to the reduced activation phenotype observed in TNs from SLE patients. To test this possibility, we stimulated TNs from SLE and healthy controls with the TLR7/8 ligand R848, G-CSF with or without GM-CSF, and the bacterial mimic fMLF. TNs from SLE patients upregulated activation markers similarly to those from controls (**Figures 1F–I**), suggesting that TNs are not exhausted and can respond to external stimuli. Similar trends were observed in patients with low and high disease activity, although differences were less pronounced in those with high disease activity (**Supplementary Figure 3**).

### 3.2 SLE traditional neutrophils exhibit a distinct phenotype at rest and following stimulation

**Figure 2:**
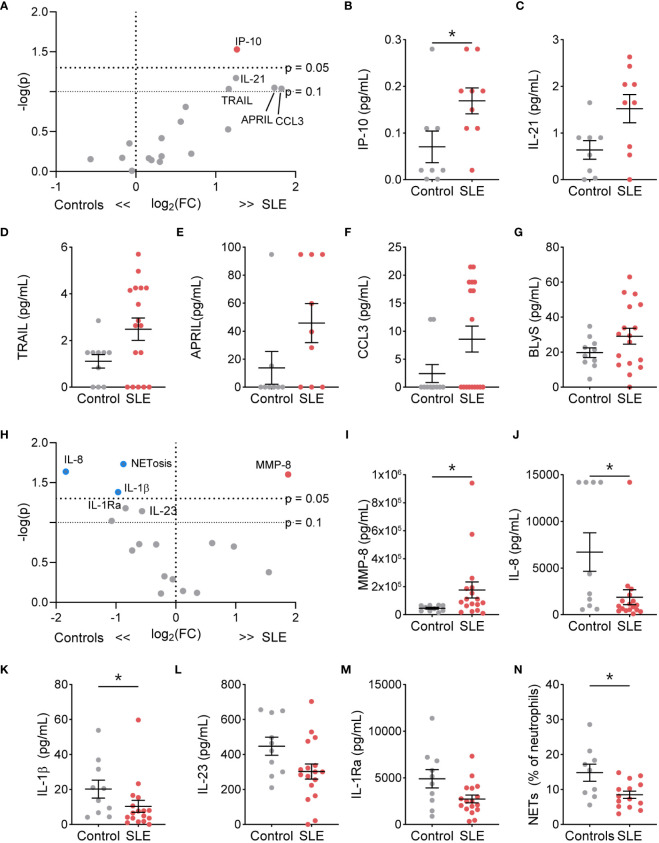
The functional phenotype of SLE traditional neutrophils differs under basal conditions and following stimulation compared to control traditional neutrophils. **(A)** Volcano plot of the difference between unstimulated functions in traditional neutrophils isolated from SLE patients and controls. Red dots indicate those significantly higher in neutrophils from SLE patients compared to those from controls. Levels of secreted **(B)** IP-10, **(C)** IL-21, **(D)** TRAIL, **(E)** APRIL, **(F)** CCL3, and **(G)** BLyS from unstimulated control or SLE traditional neutrophils as measured by flow cytometry. **(H)** Volcano plot of the difference between G-CSF+GM-CSF-stimulated functions in traditional neutrophils isolated from SLE patients and controls. Red dots indicate those significantly higher and blue dots indicate those significant lower in neutrophils from SLE patients compared to those from controls. Levels of secreted **(I)** MMP-8, **(J)** IL-8, **(K)** IL-1β, **(L)** IL-23, and **(M)** IL-1Ra from unstimulated control or SLE traditional neutrophils as measured by flow cytometry. **(N)** Percent of neutrophils with NETs was determined by microscopy following DNA and myeloperoxidase staining in G-CSF+GM-CSF-stimulated traditional neutrophils isolated from controls or SLE patients. Statistical significance was determined using a Mann-Whitney test. Lines represent mean+SEM. *p<0.05.

We next determined if TN functional phenotype, specifically NETosis, degranulation, and cytokine/chemokine production, differed from control TNs (**Supplementary Table 2**). At resting conditions, the percent of NET-forming neutrophils and release of the granule proteins lactoferrin, MMP-2, MMP-8, and MMP-9 did not differ between SLE and control TNs (**Supplementary Figure 4**). However, resting SLE TNs produced significantly more IP-10, with trends toward other increased cytokines/chemokines, such as IL-21, TRAIL, APRIL, CCL3, and BLyS, compared to control TNs (**Figures 2A–G**). In addition, TNs from patients with high disease activity released significantly less lactoferrin at rest compared to those with low disease activity and controls, while TNs from patients with low disease activity released significantly more lactoferrin compared to controls (**Supplementary Table 3**). TN functional responses did not differ based on corticosteroid usage (**Supplementary Table 4**).

Despite higher soluble mediator production at rest, SLE TNs only released more MMP-8 following stimulation with R848 (**Supplementary Figure 5**) or G-CSF and GM-CSF (**Figures 2H, I**, **Supplementary Figure 6**). Furthermore, TNs from patients with low or high disease activity released significantly more lactoferrin compared to those from controls following G-CSF and GM-CSF stimulation (**Supplementary Table 3**). In contrast, following G-CSF and GM-CSF stimulation, control TNs produced significantly more NETs, IL-8, and IL-1β, with trends towards increased IL-1Ra and IL-23, compared to SLE TNs (**Figures 2J–N**). TN functions were similar in SLE patients on or off corticosteroids (**Supplementary Table 4**).

### 3.3 SLE traditional neutrophils show differences in the expression of select miRNA

**Figure 3:**
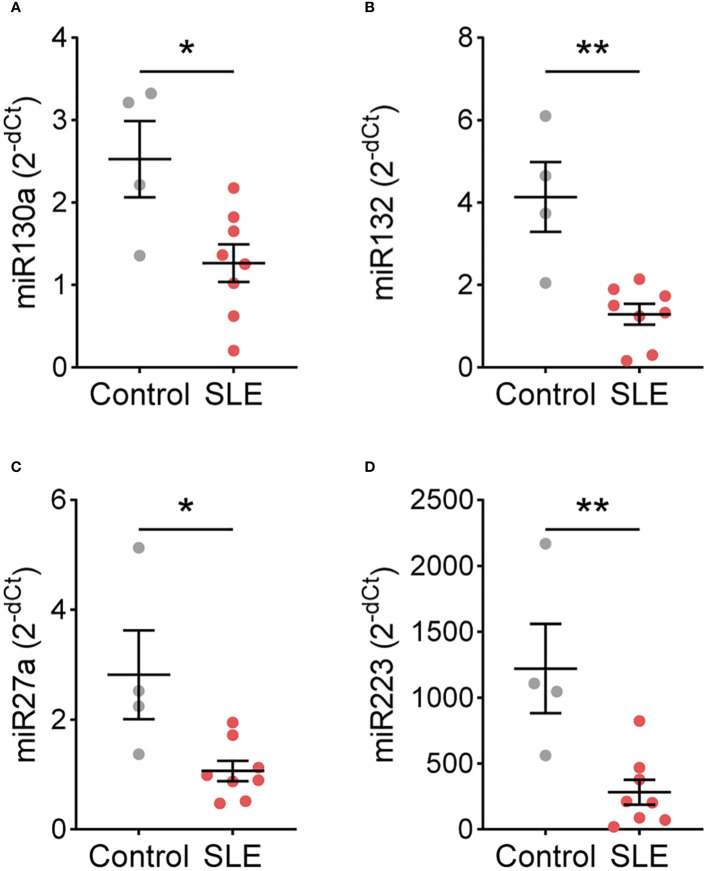
miRNA expression differs between control and SLE traditional neutrophils. Levels of **(A)** miR130a, **(B)** miR132, **(C)** miR27a, and **(D)** miR223 were measured by real-time PCR in traditional neutrophils isolated from controls or SLE patients. Statistical significance was determined using a Mann-Whitney test. Lines represent mean+SEM. *p<0.05, **p<0.01.

As SLE and control TNs displayed different functional responses, we hypothesized that gene expression may also differ. Therefore, we performed a targeted gene expression analysis for genes that differ in TNs isolated from patients with other immune-mediated diseases and healthy controls (10, 11). In a subset of patients and controls, *CSFF3, ID2, KIT, NFKBIA, NFKBIE, PIM1, TNFAIP3, TNFSF8, TRAF3*, *miR9, miR138, miR143, miR146a*, and *miR155* expression did not differ between control (n=4) and SLE (n=8) TNs (**Supplementary Figures 7**, **8**). However, the expression of *miR130a, miR132, miR27a, and miR223* was significantly higher in control TNs compared to SLE TNs (**Figure 3**).

## 4 Discussion

We found that despite reduced activation at resting, TNs from SLE patients can respond to external stimulation; however, SLE TNs exhibit different functional responses compared to control TNs. For example, resting SLE TNs produced more of the IFN-associated chemokine IP-10, which is increased right before SLE classification and flare (9, 12–14), and contributes to T cell chemoattraction and activation (15). In addition, resting SLE TNs may produce more APRIL and IL-21. Although the consequences of APRIL and IL-21 production by TNs remain unknown, previous studies have found that, in healthy individuals and during infection, neutrophils provide B cell help within the spleen through APRIL and IL-21, inducing immunoglobulin class switching, somatic hypermutation, and antibody production (10, 11). Therefore, TNs may also contribute to autoantibody production in SLE.

Interestingly, following stimulation, SLE TNs secreted less proinflammatory cytokines and NETs but released more MMP-8, while TNs from SLE patients with low or high disease activity released more lactoferrin, compared to control TNs, suggestive of increased degranulation. However, levels of the degranulation marker MMP-9 did not significantly differ. Although the role of MMP-8 in SLE is unknown, other MMPs, such as MMP-9, may play a regulatory role in SLE, potentially by cleaving autoantigens, reducing their immunogenicity and promoting immune complex clearance (16). In addition, lactoferrin may inhibit NET formation (17), consistent with the reduced NETosis we observed in SLE TNs following stimulation. Thus, SLE TNs may play a more anti-inflammatory role in the presence of G-CSF and GM-CSF, suggesting that they contribute to SLE pathogenesis differently, dependent on the inflammatory environment.

A limited number of studies have investigated the functional phenotypes of SLE compared to healthy TNs, and results have been somewhat conflicting. These studies found that at rest and following stimulation with PMA, SLE and healthy TNs exhibit similar functional responses, including ROS production, NETosis, and cytokine expression (2, 4). Consistent with these studies, we found similar CD66b expression on SLE and healthy TNs, which is a marker for granules required for ROS generation. However, in contrast to other studies, following G-CSF/GM-CSF stimulation, our data suggest that SLE TNs produce less NETs and proinflammatory cytokines compared to healthy TNs. This may be a result of different external stimulations used in our and other studies, as we did not see these differences following R848 stimulation. Consistent with functional differences, SLE and healthy TNs differ in gene expression and DNA methylation, specifically of IFN-regulated genes (2, 3, 18). Thus, our study and others demonstrate potential functional differences between SLE and healthy TNs; however, studies encompassing a more comprehensive analysis of neutrophil functional responses are needed. Although an evaluation of autologous LDGs was not included in this study, our data suggest that SLE TNs may have different functional responses compared to SLE LDGs, which are hyperresponsive and produce elevated proinflammatory cytokines and NETs compared to autologous TNs (2–4). Based on the gene expression profiles and morphology of LDGs, some studies postulate that LDGs are immature cells that are prematurely released from the bone marrow due to increased recruitment during inflammation (19), which may explain these functional differences. In addition, the increase in LDGs and excessive inflammation observed in SLE may indicate an increase in immature or exhausted SLE TNs; however, our data show that TNs express markers of mature neutrophils and retain the ability to respond to external stimuli, suggesting that SLE TNs are not immature or exhausted. Instead, we found that following stimulation, SLE TNs may be anti-inflammatory as they produce less proinflammatory cytokines and NETs. Future studies will assess whether TN functions differ based on the frequency of LDGs, providing further insight into the relationship between LDGs and TNs in SLE patients.

Our results suggest that TN responses are regulated differently in SLE compared to healthy individuals. Although the exact nature of differential regulation is unclear, we found that miRNAs implicated in neutrophil development and function (20), miR130a, miR132, miR27a, and miR223, are significantly lower in SLE neutrophils. miR130a is highly expressed in early neutrophil precursors and reduced significantly upon maturation (21); therefore, a reduction of miR-130a in SLE TNs supports the notion that SLE TNs are not more immature compared to control TNs. In contrast, miR132, miR27a, and miR223 exhibit high expression in more mature neutrophils, with a further increase of miR132 in activated neutrophils (21), suggesting that these miRNAs may instead influence the observed functional differences. For example, although the role of miR223 in neutrophils in SLE is unknown, miR223 inhibits neutrophil activation and neutrophil-driven inflammation in tuberculosis and hepatic injury mouse models (22). Thus, the reduced levels of miR223 may contribute to the increased production of proinflammatory mediators in unstimulated SLE TNs. However, future studies are needed to determine how these miRNAs contribute to TN functions in SLE.

This study was limited by the small sample size, especially in the miRNA and mRNA analyses. Furthermore, gene expression analyses were determined using targeted qPCR and the use of bulk RNA-sequencing may reveal additional differences. Finally, the SLE patients included in this study were treated and had long-standing disease, and additional differences may be revealed in populations of treatment-naïve patients.

Together, our data suggest that TNs may contribute to SLE pathogenesis; however, their contributions may differ compared to LDGs and depend on the inflammatory environment. The increased production of IP-10, APRIL, and IL-21 by SLE TNs suggest that resting SLE TNs may contribute to SLE pathogenesis through the modulation of T cell and humoral responses. In contrast, in the presence of G-CSF and GM-CSF, SLE TNs produce less inflammatory cytokines and more MMP-8 and lactoferrin, suggesting a potential regulatory role. However, future mechanistic studies are needed to determine how these functional differences impact SLE pathogenesis.

## Data availability statement

The raw data supporting the conclusions of this article will be made available by the authors, without undue reservation.

## Ethics statement

The studies involving humans were approved by Oklahoma Medical Research Foundation Institutional Review Board. The studies were conducted in accordance with the local legislation and institutional requirements. The participants provided their written informed consent to participate in this study.

## Author contributions

NJ: Conceptualization, Data curation, Formal analysis, Funding acquisition, Investigation, Methodology, Project administration, Validation, Visualization, Writing – original draft, Writing – review & editing. CW: Formal analysis, Visualization, Writing – original draft, Writing – review & editing. TA: Data curation, Investigation, Methodology, Writing – review & editing. EC: Data curation, Investigation, Methodology, Writing – review & editing. CA: Data curation, Investigation, Methodology, Writing – review & editing. JG: Data curation, Investigation, Methodology, Writing – review & editing. JJ: Conceptualization, Data curation, Formal analysis, Funding acquisition, Investigation, Methodology, Project administration, Resources, Supervision, Validation, Visualization, Writing – original draft, Writing – review & editing.
