# Supplementary material for: Neutrophils isolated from systemic lupus erythematosus patients exhibit a distinct functional phenotype
Source: Front Immunol. 2024 Mar 8;15:1339250. doi: 10.3389/fimmu.2024.1339250 (PMC10957542; doi:10.3389/fimmu.2024.1339250)
Supplement: Supplementary file 1 [file DataSheet_1.docx]

Supplementary Material

# Supplementary Tables

**Supplemental Table 1.** qPCR primers

|  | **Forward Primer** | **Reverse Primer** |
| --- | --- | --- |
| **CSF3** | CCCAGAGCCCCATGAAG | TGGCTTCCTGCACTGTC |
| **GAPDH** | GAACGGGAAGCTTGTCATCAA | ATCGCCCCACTTGATTTTGG |
| **ID2** | CTCAACACGGATATCAGCATCC | CACACAGTGCTTTGCTGTCA |
| **KIT** | GGATTCCCAGAGCCCACAA | ACATCCACTGGCAGTACAGAA |
| **NFKBIA** | CACCTCCACTCCATCCTGAA | GGTAGCCATGGATAGAGGCTAA |
| **NFKBIE** | GCTGGAAGCACTCACTTACA | CTGGGGCCTCATGAATCAC |
| **PIM1** | TGCTCAAGGACACCGTCTACA | TAGCGATGGTAGCGGATCCA |
| **TNFAIP3** | GAAGCTTGTGGCGCTGAAAA | CCTGAACGCCCCACATGTA |
| **TNFSF8** | TTCAAGAAGTCATGGGCCTACC | TGCCATCTTTGTTCCAAGACAAC |
| **TRAF3** | TGTGCAGCCCGAAGCA | ATGCTCTCTTGACACGCTGTA |
|  |  |  |

**Supplemental Table 2** Variables measured

|  | **Variables** |
| --- | --- |
| **NETosis** | % of NET-forming neutrophils |
| **Soluble mediators** | APRIL, IL-17a, IL-23, IL-1β, TNFα, BLyS, IL-1ra, IL-21, IL-27*, IP-10, TRAIL, CCL3, CXCL1, CXCL8 |
| **Granule Proteins** | Lactoferrin, MMP-2, MMP-8, MMP-9 |

*Removed from analysis

**Supplemental Table 3.** Difference in cytokine expression of unstimulated or stimulated neutrophils isolated from controls or SLE patients with high (SLEDAI >4; n=11) or low (SLEDAI<4; n=6) disease activity.

|  | Unstimulated | | | | | | G-CSF + GM-CSF | | | | | |
| --- | --- | --- | --- | --- | --- | --- | --- | --- | --- | --- | --- | --- |
|  | High SLEDAI mean (SEM) | Low SLEDAI mean (SEM) | Controls mean (SEM) | High vs Ctrl p-value | Low vs Ctrl p-value | High vs Low p-value | High SLEDAI mean (SEM) | Low SLEDAI mean (SEM) | Controls mean (SEM) | High vs Ctrl p-value | Low vs Ctrl p-value | High vs Low p-value |
| APRIL | 47.4 (47.4) | 45.3 (15.1) | 13.7 (11.7) | >0.99 | >0.99 | >0.99 | 86.6 (47) | 57.4 (17.6) | 58.8 (15) | >0.99 | >0.9999 | >0.99 |
| BLyS | 28 (10.4) | 29.7 (4.5) | 19.7 (2.8) | >0.99 | >0.99 | >0.99 | 35.1 (13.4) | 37 (5.9) | 41.4 (5.4) | >0.99 | >0.9999 | >0.99 |
| IP-10 | 0.2 (0.1) | 0.2 (0) | 0.1 (0) | >0.99 | >0.99 | >0.99 | 0.1 (0) | 0.1 (0) | 0.1 (0) | >0.99 | >0.9999 | >0.99 |
| CXCL1 | 55.3 (16.6) | 61.9 (8) | 52 (7.6) | >0.99 | >0.99 | >0.99 | 58.6 (11.5) | 106.4 (13.2) | 136.6 (30.7) | >0.99 | >0.9999 | >0.99 |
| IL-17A | 2.5 (0.6) | 0.8 (0.3) | 0.7 (0.2) | >0.99 | >0.99 | >0.99 | 1.9 (0) | 0.7 (0.2) | 0.8 (0.3) | >0.99 | >0.9999 | >0.99 |
| IL-1b | 1.3 (0.6) | 3.1 (1.1) | 3.7 (1.5) | >0.99 | >0.99 | >0.99 | 3.9 (1.2) | 13.9 (5) | 20.2 (5.2) | >0.99 | >0.9999 | >0.99 |
| IL-1ra | 973.5 (330.2) | 1393.5 (263.4) | 807.5 (120.4) | >0.99 | >0.99 | >0.99 | 1564.2 (299.2) | 3377.6 (553) | 4897.5 (989.5) | >0.99 | >0.9999 | >0.99 |
| IL-21 | 0.4 (0.4) | 1.9 (0.3) | 0.6 (0.2) | >0.99 | >0.99 | >0.99 | 1.4 (0.5) | 2.2 (0.4) | 2.5 (0.4) | >0.99 | >0.9999 | >0.99 |
| IL-23 | 58.1 (29.6) | 117.3 (31.2) | 77.1 (25.7) | >0.99 | >0.99 | >0.99 | 206.2 (48.2) | 355.6 (56.4) | 447.3 (51.7) | >0.99 | >0.9999 | >0.99 |
| CXCL8 | 98.9 (32.8) | 222.3 (56.8) | 185 (50.1) | >0.99 | >0.99 | >0.99 | 517.6 (111.2) | 2622.7 (1186.7) | 6714.8 (2063.4) | >0.99 | >0.9999 | >0.99 |
| Lactoferrin | 408102.8 (114445.9) | 1665806 (375656.5) | 1375678.7 (351310.9) | **<0.001** | **0.04** | **<0.001** | 3869626.8 (2362589.2) | 4373943.3 (1390154) | 1351604.9 (239069.4) | **<0.001** | **<0.0001** | 0.60 |
| CCL3 | 8.7 (4.1) | 8.5 (3) | 2.4 (1.6) | >0.99 | >0.99 | >0.99 | 25.1 (3.6) | 34.5 (4.5) | 65.5 (26.9) | >0.99 | >0.9999 | >0.99 |
| MMP-2 | 4030.9 (330) | 10564.1 (1497.7) | 7283.4 (1245.3) | 0.99 | 0.99 | 0.99 | 9919.3 (2500.1) | 14376.2 (3129.4) | 8815.4 (993.8) | >0.9999 | >0.9999 | >0.99 |
| MMP-8 | 34532.6 (12841.1) | 78770.5 (16531.6) | 51268 (11451.2) | 0.99 | 0.97 | 0.95 | 110402.2 (40156.4) | 212802.3 (86179.9) | 45922.7 (6464.2) | 0.992 | 0.9279 | 0.98 |
| MMP-9 | 109972.8 (38600.9) | 265796.3 (51727.3) | 223067.8 (33086.8) | 0.70 | 0.93 | 0.5 | 293729.4 (48529.4) | 784999.8 (300593) | 301861.7 (54635) | 0.9999 | 0.5352 | 0.62 |
| TNFa | 0.2 (0.2) | 0.4 (0.1) | 0.2 (0.1) | >0.99 | >0.99 | >0.99 | 0.2 (0.2) | 0.6 (0.1) | 0.8 (0.2) | >0.9999 | >0.9999 | >0.99 |
| TRAIL | 2.1 (0.8) | 2.7 (0.6) | 1.1 (0.3) | >0.99 | >0.99 | >0.99 | 2.7 (1.4) | 2.4 (0.6) | 2.6 (0.5) | >0.9999 | >0.9999 | >0.99 |
| NETosis | 5.7 (1.5) | 4.1 (0.7) | 4.2 (1) | >0.99 | >0.99 | >0.99 | 6.9 (1.7) | 8.9 (1.3) | 14.8 (2.5) | >0.9999 | >0.9999 | >0.99 |

p-values determined using a 2-way ANOVA with Tukey’s multiple comparisons test.

**Supplemental Table 4.** Difference in cytokine expression of unstimulated or stimulated neutrophils isolated from SLE patients on corticosteroids (CS; n=6) or off CS (no CS; n=11).

|  | **Unstimulated** | | | **G-CSF + GM-CSF** | | |
| --- | --- | --- | --- | --- | --- | --- |
|  | **No CS mean (SEM)** | **CS mean (SEM)** | **p-value** | **No CS mean (SEM)** | **CS mean (SEM)** | **p-value** |
| **APRIL** | 46.3 (17.8) | 17.8 (6) | >0.9999 | 46.4 (98.9) | 18.9 (20.1) | 0.2143 |
| **BLyS** | 25.5 (4.9) | 4.9 (11) | 0.4474 | 33.1 (42.3) | 8 (7.8) | 0.3375 |
| **IP-10** | 0.2 (0.03) | 0.03 (6) | 0.2381 | 0.1 (0.1) | 0.02 (0.03) | 0.619 |
| **CXCL1** | 50.8 (8.5) | 8.5 (11) | 0.2447 | 76 (114.3) | 12.5 (17.5) | 0.149 |
| **IL-17A** | 1.6 (0.4) | 0.4 (6) | 0.119 | 1.1 (0.6) | 0.2 (0.6) | 0.381 |
| **IL-1b** | 1.7 (0.5) | 0.5 (11) | 0.3615 | 7.1 (16.4) | 2.2 (8.9) | 0.3125 |
| **IL-1ra** | 917.1 (172.1) | 172.1 (11) | 0.0782 | 2286.7 (3564.2) | 434.9 (855.4) | 0.3011 |
| **IL-21** | 1.6 (0.4) | 0.4 (6) | 0.6667 | 1.9 (2.3) | 0.3 (1) | 0.8452 |
| **IL-23** | 115.5 (33.3) | 33.3 (11) | 0.4709 | 269.2 (364.5) | 49.4 (82) | 0.5739 |
| **IL-8** | 146.7 (33.2) | 33.2 (11) | 0.7325 | 1170.5 (3179.9) | 304.1 (2210.8) | 0.5908 |
| **Lactoferrin** | 1036906.6 (214890.5) | 214890.5 (11) | 0.9612 | 4159215.4 (4263294.7) | 1363461.9 (2425456.4) | 0.5095 |
| **CCL3** | 6.6 (2.8) | 2.8 (11) | 0.3174 | 29 (35.2) | 4.2 (5.4) | 0.4779 |
| **MMP-2** | 8162.5 (1914.2) | 1914.2 (6) | 0.3452 | 11484.3 (17188.8) | 1466.6 (7361.1) | 0.7143 |
| **MMP-8** | 58657.9 (17255) | 17255 (11) | 0.3502 | 180824.2 (169028.7) | 79395.5 (84085.7) | 0.8836 |
| **MMP-9** | 166367.2 (35406.1) | 35406.1 (11) | 0.3502 | 422968.4 (957453.7) | 70358.2 (556572.9) | 0.8836 |
| **TNFa** | 0.4 (0.1) | 0.1 (6) | 0.4524 | 0.3 (0.8) | 0.1 (0.2) | 0.119 |
| **TRAIL** | 2.1 (0.6) | 0.6 (11) | 0.3286 | 2.6 (2.4) | 0.8 (1) | 0.8652 |
| **NETosis** | 5.3 (1.1) | 3.9 (0.7) | 0.6889 | 7.7 (1.3) | 8.7 (1.8) | 0.6889 |

*p-values determined using a Mann-Whitney test

# Supplementary Figures


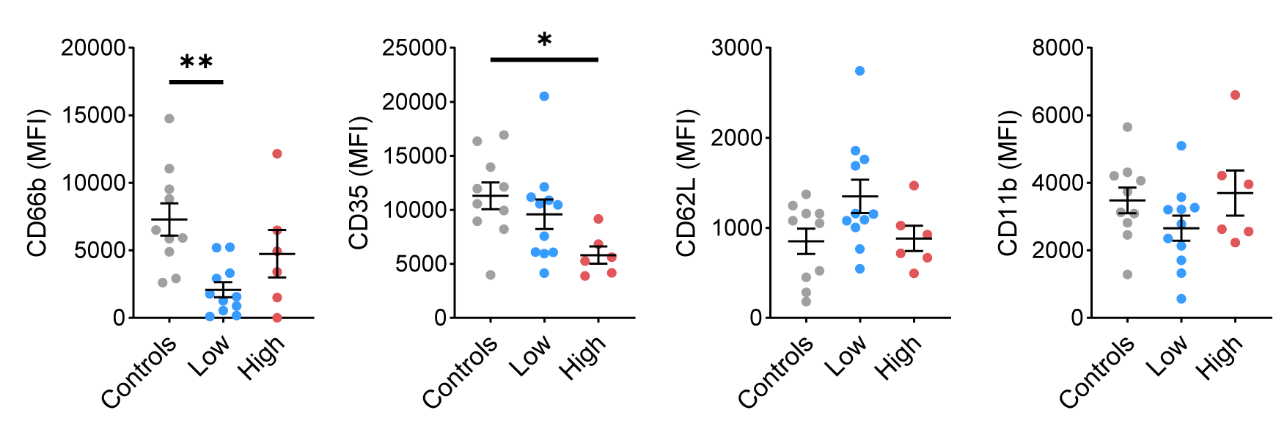
**Supplemental Figure 1. Neutrophil activation based on SLE disease activity.** Expression of neutrophil activation makers was determined on traditional neutrophils isolated from controls or SLE patients with low (SLEDAI <4; n=11) or high (SLEDAI >4; n=6) disease activity using flow cytometry. Statistical significance was determined using a one-way ANOVA with Tukey’s multiple comparisons test. Lines indicate mean­+SEM. *p<0.05, **p<0.01.


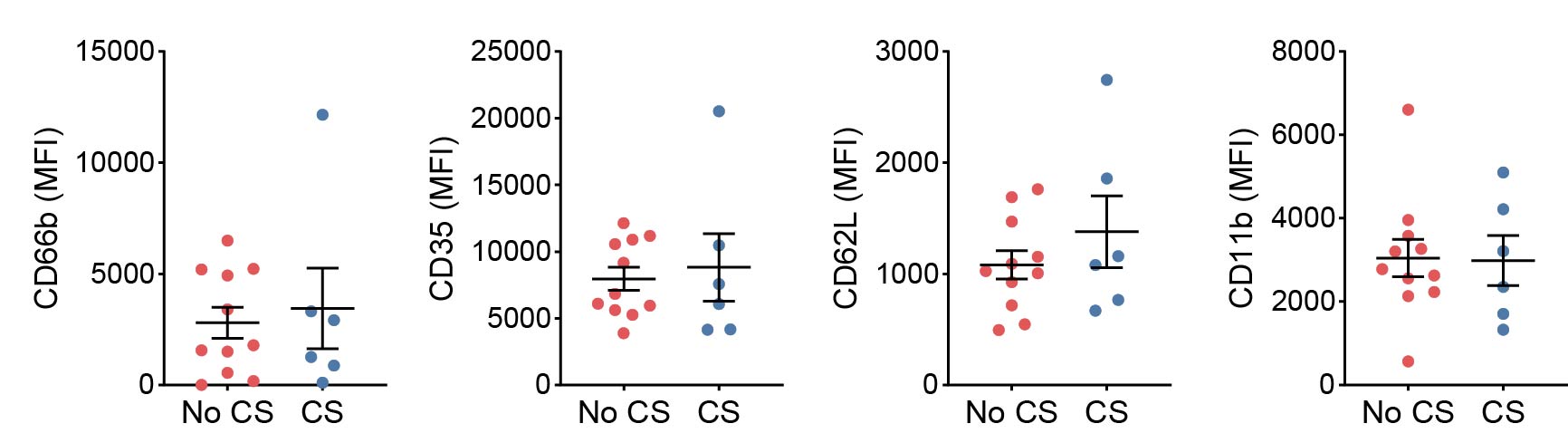


**Supplemental Figure 2. Neutrophil activation does not differ in SLE patients based on corticosteroid (CS) usage.** Expression of neutrophil activation makers was determined on traditional neutrophils isolated from SLE patients on (CS; n=6) or off (no CS; n=11) CSs using flow cytometry. Statistical significance was determined using a Mann-Whitney test. Lines indicate mean­+SEM.


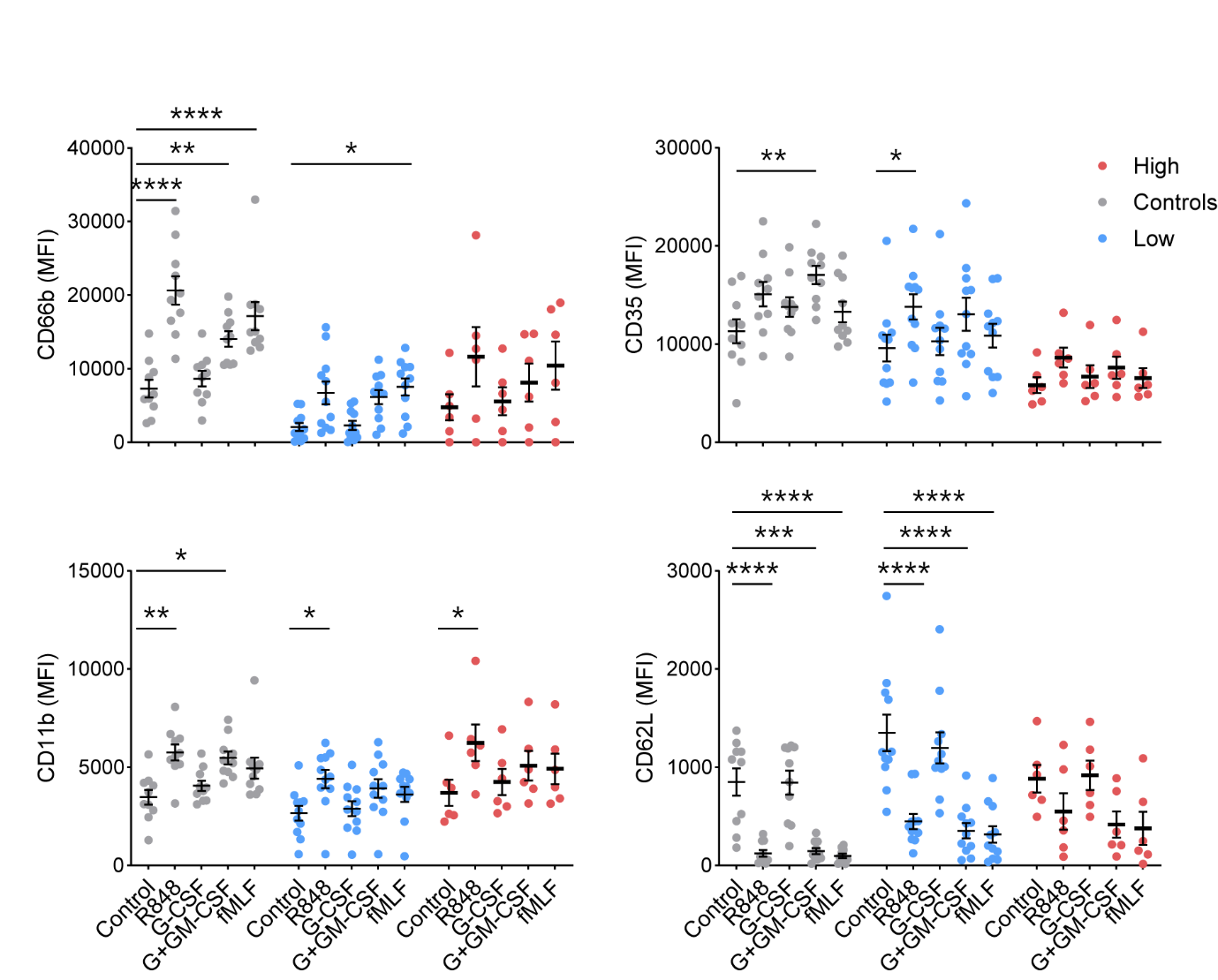


**Supplemental Figure 3.** **Neutrophil activation following stimulation based on SLE disease activity.** CD66b, CD35, CD11b, and CD62L expression was determined on unstimulated or stimulated traditional neutrophils isolated from controls or SLE patients with low (SLEDAI <4; n=11) or high (SLEDAI >4; n=6) disease activity using flow cytometry. Statistical significance was determined using a 2-way ANOVA with Sidak’s multiple comparisons test. Lines indicate mean+SEM. *p<0.05, **p<0.01, ***p<0.001, ****p<0.0001.

**
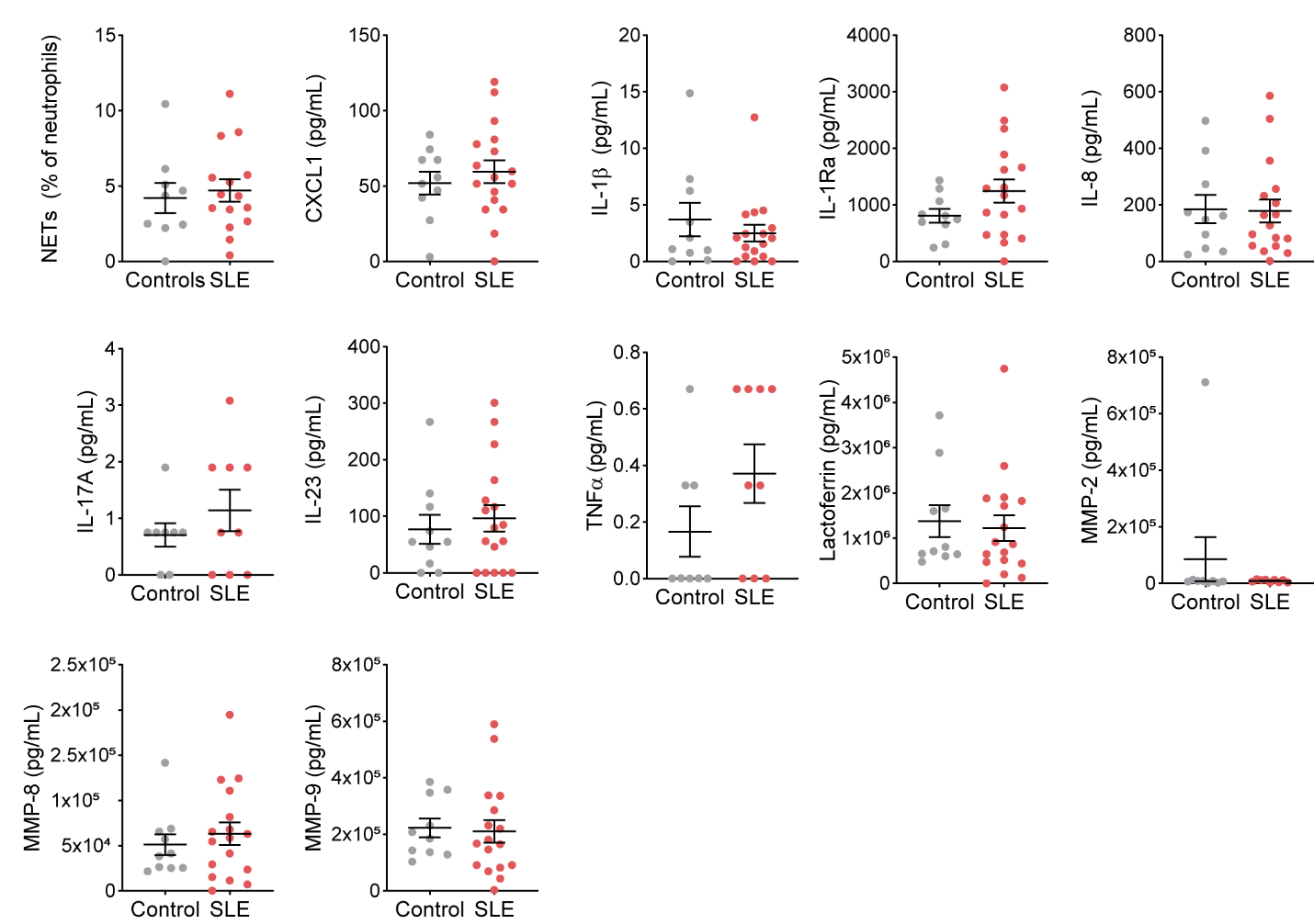
**

**Supplemental Figure 4. Effector functions of unstimulated traditional neutrophils isolated from controls or SLE patients.** Percent of neutrophils producing NETs or levels of secreted soluble mediators was determined using microscopy or flow cytometry, respectively, in unstimulated traditional neutrophils isolated from controls or SLE patients. Statistical significance was determined using a Mann-Whitney test. Lines indicate mean+SEM.

**
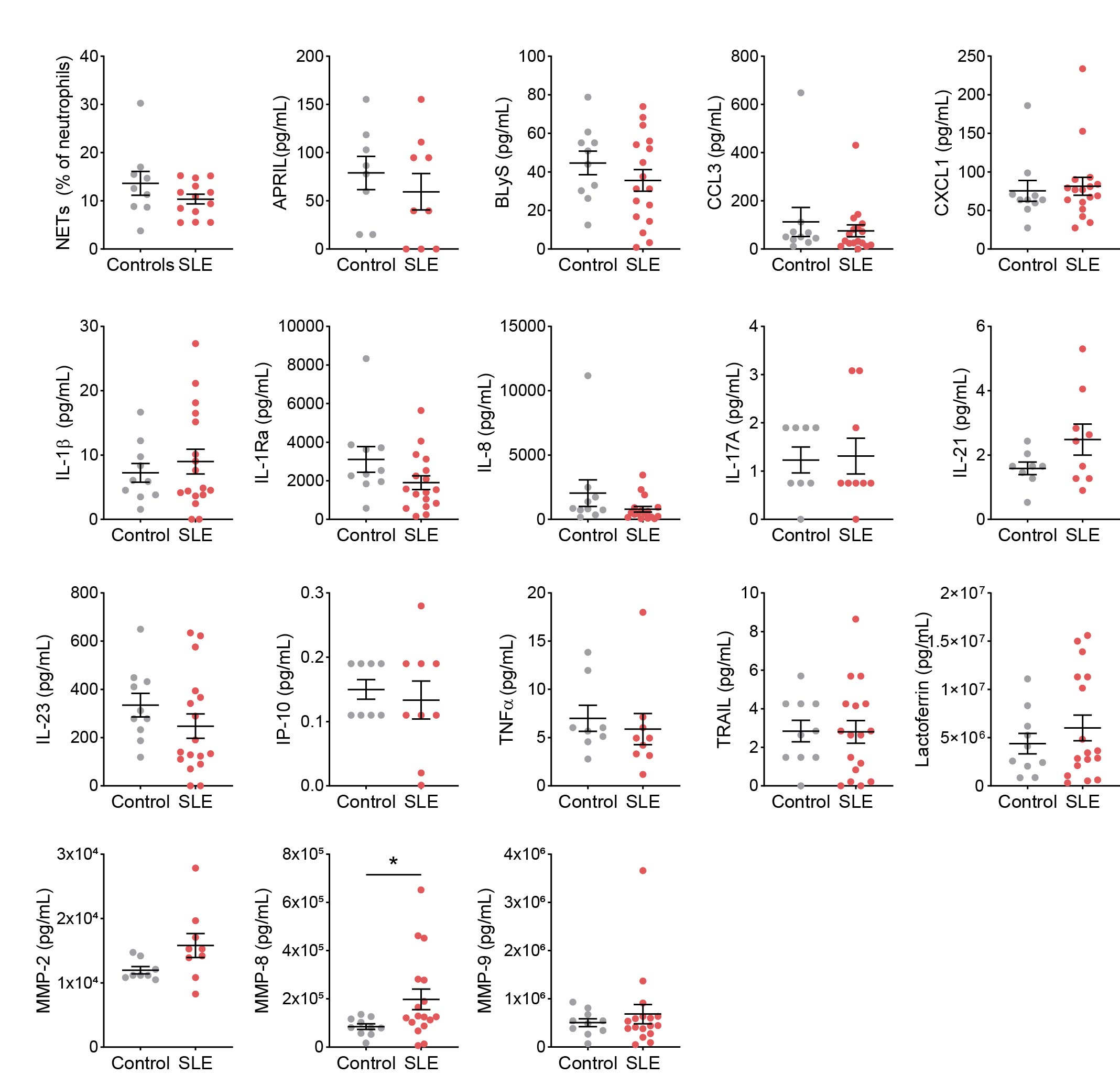
**

**Supplemental Figure 5. Effector functions of R848-stimulated traditional neutrophils isolated from controls or SLE patients.** Percent of neutrophils producing NETs or levels of secreted soluble mediators was determined using microscopy or flow cytometry, respectively, in R848-stimulated traditional neutrophils isolated from controls or SLE patients. Statistical significance was determined using a Mann-Whitney test. Lines indicate mean+SEM.

**
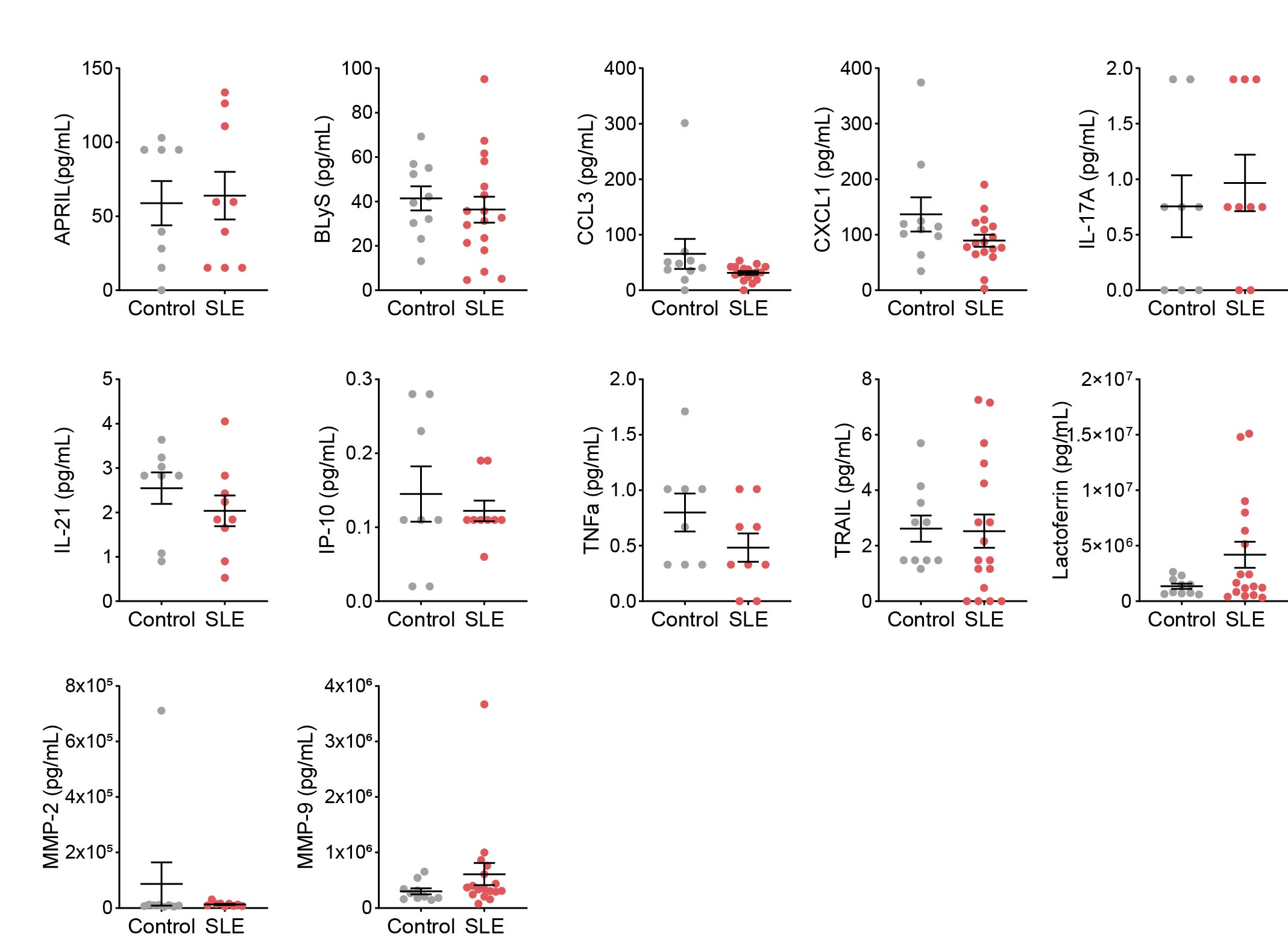
**

**Supplemental Figure 6. Effector functions of G-CSF/GM-CSF-stimulated traditional neutrophils isolated from controls or SLE patients.** Levels of secreted soluble mediators was determined using flow cytometry in G-CSF+GM-CSF-stimulated traditional neutrophils isolated from controls or SLE patients. Statistical significance was determined using a Mann-Whitney test. Lines indicate mean+SEM.

**
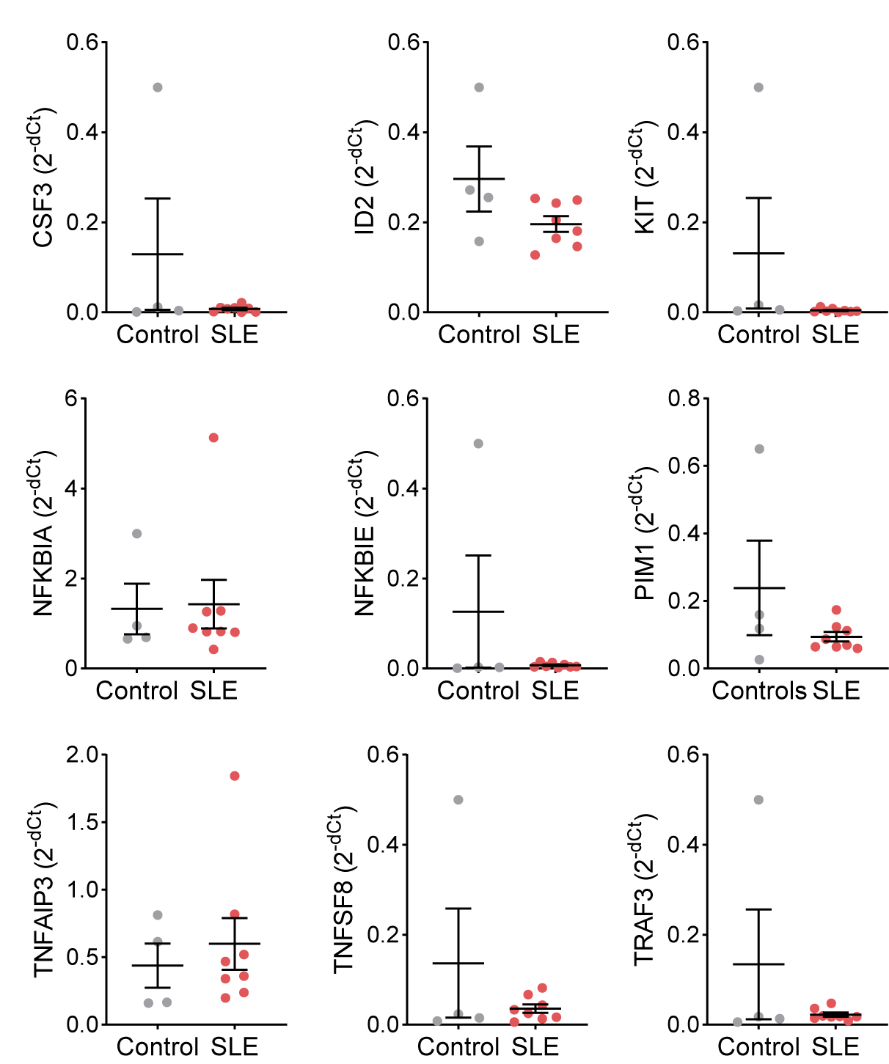
**

**Supplemental Figure 7. mRNA expression in traditional neutrophils isolated from controls or SLE patients.** mRNA expression was measured by real-time PCR in traditional neutrophils isolated from controls or SLE patients. Statistical significance was determined using a Mann-Whitney test. Lines represent mean+SEM.

**
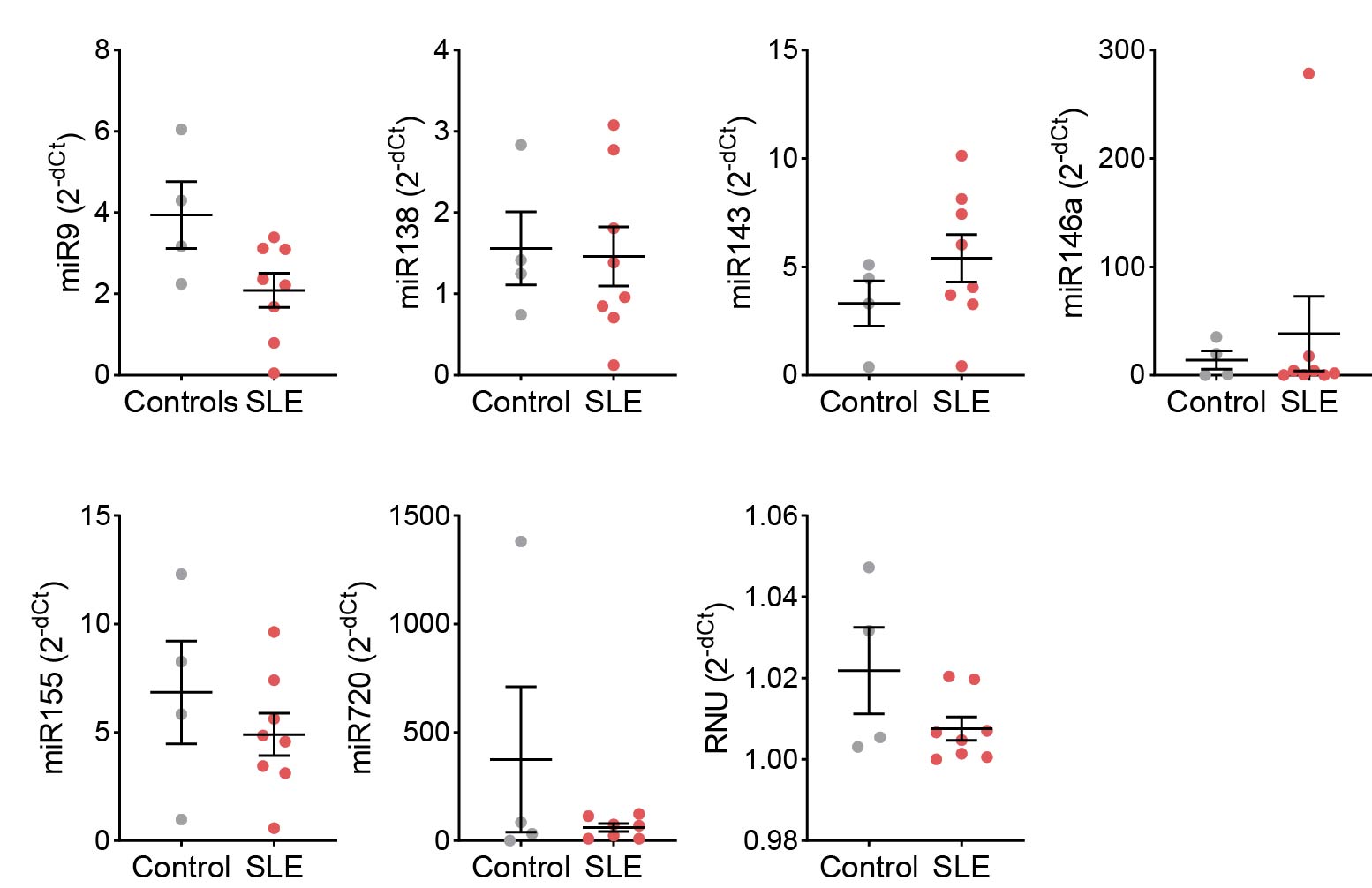
**

**Supplemental Figure 8. miRNA expression in traditional neutrophils isolated from controls or SLE patients.** miRNA expression was measured by real-time PCR in traditional neutrophils isolated from controls or SLE patients. Statistical significance was determined using a Mann-Whitney test. Lines represent mean+SEM.
